# Methyl-CpG-binding (SmMBD2/3) and chromobox (SmCBX) proteins are required for neoblast proliferation and oviposition in the parasitic blood fluke *Schistosoma mansoni*

**DOI:** 10.1371/journal.ppat.1007107

**Published:** 2018-06-28

**Authors:** Kathrin K. Geyer, Sabrina E. Munshi, Helen L. Whiteland, Narcis Fernandez-Fuentes, Dylan W. Phillips, Karl F. Hoffmann

**Affiliations:** Institute of Biological, Environmental and Rural Sciences, Aberystwyth University, Aberystwyth, United Kingdom; George Washington University, UNITED STATES

## Abstract

While schistosomiasis remains a significant health problem in low to middle income countries, it also represents a recently recognised threat to more economically-developed regions. Until a vaccine is developed, this neglected infectious disease is primarily controlled by praziquantel, a drug with a currently unknown mechanism of action. By further elucidating how *Schistosoma* molecular components cooperate to regulate parasite developmental processes, next generation targets will be identified. Here, we continue our studies on schistosome epigenetic participants and characterise the function of a DNA methylation reader, the *Schistosoma mansoni* methyl-CpG-binding domain protein (SmMBD2/3). Firstly, we demonstrate that SmMBD2/3 contains amino acid features essential for 5-methyl cytosine (5mC) binding and illustrate that adult schistosome nuclear extracts (females > males) contain this activity. We subsequently show that SmMBD2/3 translocates into nuclear compartments of transfected murine NIH-3T3 fibroblasts and recombinant SmMBD2/3 exhibits 5mC binding activity. Secondly, using a yeast-two hybrid (Y2H) screen, we show that SmMBD2/3 interacts with the chromo shadow domain (CSD) of an epigenetic adaptor, *S*. *mansoni* chromobox protein (SmCBX). Moreover, fluorescent *in situ* hybridisation (FISH) mediated co-localisation of *Smmbd2/3* and *Smcbx* to mesenchymal cells as well as somatic- and reproductive- stem cells confirms the Y2H results and demonstrates that these interacting partners are ubiquitously expressed and found within both differentiated as well as proliferating cells. Finally, using RNA interference, we reveal that depletion of *Smmbd2/3* or *Smcbx* in adult females leads to significant reductions (46–58%) in the number of proliferating somatic stem cells (PSCs or neoblasts) as well as in the quantity of *in vitro* laid eggs. Collectively, these results further expand upon the schistosome components involved in epigenetic processes and suggest that pharmacological inhibition of SmMBD2/3 and/or SmCBX biology could prove useful in the development of future schistosomiasis control strategies.

## Author summary

Schistosomiasis, caused by infection with blood fluke worms, is responsible for chronic disability and debilitating pathology in millions of infected individuals living in deprived regions of the developing world. Currently, schistosomiasis is primarily controlled by administration of a single drug (praziquantel) with a currently unknown mechanism of action and an inability to prevent reinfection or kill juvenile blood flukes. Therefore, to limit the spread and lower the global prevalence of this neglected infectious disease, praziquantel replacement or alternative strategies are urgently needed. One such strategy is to identify molecular targets essential for schistosome biology and to characterise how their loss of function affects parasite developmental processes. By doing so, new drug targets or vaccine candidates can be progressed. Here, we extend our work on the characterisation of *Schistosoma mansoni* epigenetic processes and reveal that two interacting components (*S*. *mansoni* methyl-CpG-binding domain protein, SmMBD2/3 and *S*. *mansoni* chromobox protein, SmCBX) are instrumental for maintaining the proliferative capacity of the single most important parasite cell population—proliferating schistosome stem cells (or neoblasts). We additionally demonstrate that these two proteins are necessary for maintaining schistosome egg production, a lifecycle feature responsible for human pathology and disease transmission. Developing drugs that disrupt the interaction of these epigenetic participants or inhibit their activity could highlight a novel approach for controlling schistosomiasis.

## Introduction

Characterised by a complex lifecycle alternating between two different hosts (snail and mammal) and a fresh water ecosystem, schistosomes are highly evolved human pathogens responsible for the neglected infectious disease schistosomiasis. Predominantly found in sub-tropical and tropical regions of resource-poor communities, schistosomiasis kills thousands of individuals per year and causes chronic disability in millions more [1]. Until a immunoprophylactic vaccine can be developed, existing treatment relies on chemotherapeutic administration of praziquantel (PZQ) to individuals living in endemic communities [2]. Use of a single anti-parasitic drug with a currently unknown mechanism of action (perhaps acting as a G-protein coupled receptor agonist [3]) and limited efficacy against juvenile schistosomes [4], however, raises serious concerns in meeting the ambitious targets set by the World Health Organisation for achieving schistosomiasis elimination in selected regions and countries by 2020 [5]. Therefore, furthering our understanding into how schistosomes respond to diverse environmental stimuli (water, snail or human) may simultaneously reveal the molecular processes essential for lifecycle transmission as well as the specific components suitable for next-generation anti-schistosomal drug or vaccine development. Epigenetic processes that shape chromatin modifications as well as regulate both heritable and environmentally influenced phenotypes present a rich molecular area in which to identify these key schistosome components [6].

While others have explored the role of histone modifying enzymes (HMEs) [7] in mammalian host infection, cercariae to schistosomula transformation, parasite viability, egg production and sexual differentiation of adults [8–13], we have investigated the epigenetic biology of the core schistosome DNA methylation machinery components DNA methyltransferase 2 (SmDNMT2) and methyl-CpG binding domain protein (SmMBD2/3) [14]. After first demonstrating that the co-regulated expression of *Smdnmt2* and *Smmbd2/3* throughout the schistosome lifecycle mirrored the abundance of DNA methylation, we provided important evidence that SmDNMT2 is, indeed, a functional DNA methyltransferase. In this previous study, RNA interference (RNAi) suppression of *Smdnmt2* led to a significant decrease in global DNA methylation in adult schistosomes. Together with 5-azacytidine (5-AzaC) mediated inhibition of adult worm DNA methylation, egg production, embryo maturation and ovarian development, this functional genomics datum strongly suggested an important, but enigmatic [15], role for DNA methylation (and SmDNMT2 activity) in schistosome biology and oviposition. However, in depth functional analysis of the DNA methylation reader, SmMBD2/3, was not fully explored in our previous study [14]. As methyl-CpG binding domain (MBD) proteins importantly link DNA methylation to higher order chromatin structures [16], SmMBD2/3 characterisation could provide further insight into the downstream action of an intact DNA methylation machinery in this parasite.

Despite the diversity of MBD family members found within vertebrates (MBDs 1–6 and MeCP2), invertebrate genomes generally only contain one ancestral form, MBD2/3 [16, 17]. Within the invertebrates, the most comprehensive functional data for MBD2/3 proteins have been obtained from *Drosophila melanogaster* (dMBD2/3), largely due to its unusual mCpT/A-binding capability [18]. Non-CpG methylation has been associated particularly with DNMT2 activity [14, 15, 19], and so it appears that dMBD2/3 has evolved to adapt to these conditions in this DNMT2-only organism. Amongst the Platyhelminthes, only MBD2/3 from the planarian *Schmidtea mediterranea* (SmedMBD2/3) has been characterised to date [20]. An arginine to lysine mutation at position 17, which is known to interact directly with mCpGs [21], is likely to render SmedMBD2/3 incapable of binding to methylated DNA. Interestingly, *Smedmbd2/3* expression was exclusively found in adult somatic stem cells (ASCs), called neoblasts, as well as germline cells, and was deemed essential for their differentiation during tissue regeneration and homeostasis. RNAi-mediated knockdown of *Smedmbd2/3* resulted in a failure to correctly regenerate several organs, including the eyes, gut and pharynx. This observation, combined with a lack of detectable DNA methylation, suggests an entirely DNA methylation-independent role for SmedMBD2/3 in this non-parasitic platyhelminth species.

Due to its currently unknown role in schistosome molecular or epigenetic processes, we herein have conducted a thorough investigation of the first parasitic platyhelminth MBD2/3, SmMBD2/3. Using a combination of experimental approaches, we demonstrated that SmMBD2/3 is a nuclear localised, functional 5-methyl cytosine (5mC) binding protein capable of interacting with an epigenetic adaptor protein—*S*. *mansoni* chromobox protein (SmCBX). Functional genomics-led analyses of SmMBD2/3 and SmCBX have further indicated that both gene products are required for schistosome neoblast proliferation and oviposition. Together, our data provide additional roles for ancestral MBD2/3 function in DNMT2-only organisms and highlight the SmMBD2/3-SmCBX protein complex as a novel target for combating schistosomiasis.

## Materials and methods

### Ethics statement

All mouse procedures performed at Aberystwyth University (AU) adhered to the United Kingdom Home Office Animals (Scientific Procedures) Act of 1986 (project license PPL 40/3700) as well as the European Union Animals Directive 2010/63/EU and were approved by AU’s Animal Welfare and Ethical Review Body (AWERB). In adherence to the Animal Welfare Act and the Public Health Service Policy on Humane Care and Use of Laboratory Animals, all mouse procedures performed at UT Southwestern Medical Center were approved by the Institutional Animal Care and Use Committee (IACUC) of the UT Southwestern Medical Center (protocol approval number APN 2014–0072).

### Parasite material

A Puerto Rican strain (NMRI) of *Schistosoma mansoni* was used throughout the study and passaged between *Mus musculus* (Tuck Ordinary; TO) and *Biomphalaria glabrata* (NMRI albino and pigmented hybrid [22]) hosts. Cercariae were shed from both *B*. *glabrata* strains by exposure to light in an artificially heated room (26 ^o^C) for 1 hr and used to percutaneously infect *M*. *musculus* (200 cercariae/mouse) [23]. Adult schistosomes were obtained from *M*. *musculus* at 7 wks post-infection and used for RNA interference (RNAi), fluorescence *in situ* hybridization (FISH) and for the generation of nuclear protein extracts.

### Multiple sequence alignment

Comparison of methyl-CpG binding domains (MBDs, PF01429) from multiple MBD family members was performed by multiple sequence alignment, generated using MUSCLE v3.8 [24]. The NCBI accession numbers of these MBD family members comprise: *Apis mellifera* MBD1, XP_003250634.1; *Homo sapiens* MBD1, NP_002375.1; *M*. *musculus* MBD1, NP_038622.2; *H*. *sapiens* MeCP2, NP_001104262.1; *M*. *musculus* MeCP2, NP_001075448.1; *Xenopus laevis* MeCP2, NP_001081854.1; *H*. *sapiens* MBD4, NP_001263201.1; *M*. *musculus* MBD4, NP_034904.2; *H*. *sapiens* MBD2, NP_003918.1; *M*. *musculus* MBD2, NP034903.2; *Gallus gallus* MBD2, NP_001012403.1; *H*. *sapiens* MBD3, NP_001268382.1; *M*. *musculus* MBD3, NP_038623.1; *X*. *laevis* MBD3, AAD55389.1; *Bombyx mori* MBD2/3, XP_004929675.1; *Ancylolomia pectinifera* MBD2/3, ACF05483.1; *Hemicentrotus pulcherrimus* MBD2/3, ACF05485.1; *S*. *mansoni* MBD2/3, CCD59176.1; *Drosophila melanogaster* MBD2/3, NP_731370.1. For *Acheta domesticus* MBD2/3, no NCBI accession number could be located; therefore, the amino acid sequence for this family member was retrieved from Uno *et al*. [25]. The sequences were inspected for homology and the presence of conserved and semi-conserved residues. Residues that directly bind to the DNA backbone or 5mC decorated dinucleotides in the published solution structures [21, 26] were manually annotated, along with residues previously shown to result in reduced 5mC-binding after mutagenesis.

### Homology modeling of SmMBD2/3 with a 5mC containing DNA complex

The three-dimensional structure of the MBD within SmMBD2/3 (GenBank ID: AEK05283.1) was derived by homology modelling using M4T [27, 28]. The MBD template selected for SmMBD2/3 modelling was the three-dimensional structure of *G*. *gallus* MBD2 (Protein Databank [29] identification code: 2KY8) [26]. The sequence identity between SmMBD2/3 and the chicken MBD2 template was 42% with a sequence coverage over 90%, hence well within the acceptable range for comparative modelling techniques [30]. The quality and stereochemistry of the SmMBD2/3-5mC model was assessed using Prosa-II [31] and PROCHECK [32] respectively.

### Cell culture and transfection

Full-length cDNAs encoding SmMBD2/3 (HM991455.1) and red fluorescent protein (RFP) were cloned into the pkFLAG vector and expressed in NIH-3T3 cells (ECACC number 86041101) with a C-terminal FLAG tag (SmMBD2/3-FLAG and RFP-FLAG). Briefly, NIH-3T3 fibroblasts were cultured at 37°C in a 5% CO_2_ environment in DMEM supplemented with penicillin (100 U/ml), streptomycin (100 μg/ml), 10% v/v new born calf serum (Sigma Aldrich) and 2 mM L-glutamine. At 24 hr prior to transfection, 50,000 cells were seeded per chamber slide well (1.7cm^2^) to give 70–90% confluency the next day. Cells were transfected with either 1 μg of pkFLAG-RFP or pkFLAG-SmMBD2/3 per well of a chamber slide using 2 μl of Turbofect transfection reagent (Thermo Scientific) according to the manufacturer’s instructions. For negative transfection controls, an equal volume of water was used in replacement of plasmid. Transfections proceeded for 24 hr before preparation of slides for microscopy.

### Transfected NIH-3T3 immunoassaying and visualisation

For SmMBD2/3-FLAG immunolocalisation, cells were fixed in 5% v/v formaldehyde solution (5 min), washed 3 X 5 min with PBS, permeabilised with 0.15% v/v Triton X-100 (2 min) and washed again with PBS (3 X 5 min). Slides were then incubated for 1 hr with a 1:400 dilution (PBS/1% BSA) of anti-FLAG primary antibody (M2 clone, F3165, raised in mice, Sigma Aldrich) and washed once in PBS/1% BSA for 5 min before secondary antibody incubation. Here, Alexa Fluor 488 (F(ab’)2 fragment, anti-mouse IgG (H + L), raised in goat) conjugated Abs (1:200 dilution in PBS/10% v/v new born calf serum) were added to the slides and incubated for 1 hr. Slides were finally washed 3 X 5 min in PBS. For ‘water’ transfected cells (negative control), staining was also performed in this manner. For RFP-FLAG transfected cells, only the initial formaldehyde fixation step and PBS wash was required.

Prior to visualisation, cells were immersed in mounting solution (Vectashield, Vector Labs) supplemented with DAPI (4',6-diamidino-2-phenylindole, 1.5 μg/ml). Fluorescent microscopic images were captured on a Leica TCS SP5II laser scanning confocal microscope (LSCM) fitted with a 63 X (oil immersion) objective (2.3 X zoom) using the Leica LAS-AF software. Green (SmMBD2/3-FLAG) and red (RFP-FLAG) fluorescence were visualised with an argon or diode-pumped, solid state (DPSS) laser at 488 nm and 561 nm, respectively. DAPI was visualised using a 405 nm blue diode laser. Obtained images were deconvolved using AutoQuant X2 (Media Cybernetics) and analysed in Imaris 7.3 (Bitplane).

### Quantification of SmMBD2/3 subcellular localisation

ImageJ was used for quantitative analysis of immunofluorescence localisation of SmMBD2/3-FLAG and RFP-FLAG in NIH-3T3 cells. The microscopic fields of view selected for image analysis contained a total of approximately 50 cells (~25% of which were transfected). Every transfected cell within each image was used in the analysis, counting top to bottom, to ensure no subjective bias could influence the selection. A total of 40 transfected cells were analysed across three images for both pkFLAG-SmMBD2/3 and pkFLAG-RFP constructs. The total pixel intensity (derived from green or red fluorescence produced by SmMBD2/3-FLAG or RFP-FLAG, respectively) was measured for manually annotated nuclear and whole cell regions in ImageJ. Nuclear regions were annotated according to DAPI staining. No background subtraction of values was required, because pixel intensity readings for the surrounding area (verified individually for each image) were zero. Nuclear fluorescence readings were normalised as a percentage of the total cell fluorescence to account for variation in whole image fluorescence intensity, varying cell size and variable levels of protein expression within each cell. Nuclear and cytosolic fluorescence values were analysed and compared by one-way ANOVA and Tukey’s Honest Significant Difference (HSD) test to confirm statistical significance.

### Recombinant SmMBD2/3 expression in *Escherichia coli*

Full-length SmMBD2/3 (HM991455.1) was cloned into the pET30a vector (Novagen, UK) and expressed in One Shot BL21 (DE3) *E*. *coli* competent cells (Invitrogen, UK) to contain a C-terminal poly-histidine tag (His_6_) as previously described for *S*. *mansoni* venom allergen like 9 (SmVAL9) [33]. Briefly, isopropyl β-D-1-thiogalactopyranoside (IPTG) induced bacterial cell pellets were resuspended in 15 ml of lysis buffer (50 mM NaH_2_PO_4_, 300 mM NaCl, 10 mM imidazole (IMDZ) + protease inhibitors (cOmplete, mini, EDTA-free tablets, Roche)) and lysed in a Cell Disruption System (Constant Systems) at 30,000 Psi. The lysates were centrifuged at 21,000 x *g*, 4°C for 20 min to yield the soluble protein fraction. The soluble protein fraction was passaged 3 x over a column containing 500 μl Ni-NTA agarose beads (Qiagen). Purification of recombinant (r) SmMBD2/3 was achieved using wash buffers (50 mM NaH_2_PO_4_, 300 mM NaCl, + protease inhibitors (cOmplete, mini, EDTA-free tablets, Roche)) containing increasing concentrations of IMDZ. An initial 40 mM IMDZ wash buffer was used (30 ml per 800 ml culture) followed by a 100 mM IMDZ wash (10 ml). rSmMBD2/3 elution was achieved using 10 ml wash buffer containing 250 mM IMDZ. For the “un-induced” negative control protein sample, bacteria were processed identically, except the IPTG induction step was omitted. The resulting cell pellets were subjected to the purification scheme used for IPTG-induced rSmMBD2/3. This process produced a soluble protein fraction that was enriched for the Ni-NTA co-purifying *E*. *coli* products present in the purified rSmMBD2/3 sample. Western blot analysis of purified rSmMBD2/3 and “un-induced’ Ni-NTA co-purifying *E*. *coli* products was performed essentially as described [33]. The HisProbe-Horseradish peroxidase (HRP) conjugate (ThermoScientific) used to detect rSmMBD2/3- His_6_ was used at a 1:4000 dilution.

### Mass spectrometry analysis of recombinant SmMBD2/3

Amino acid sequence evaluation of recombinant SmMBD2/3 (rSmMBD2/3) was confirmed by matrix assisted laser desorption ionisation time of flight (MALDI-TOF) mass spectrometry. The protein band corresponding to rSmMBD2/3-His_6_ (36.5 kDa) was excised from Coomassie blue stained polyacrylamide gels and subjected to in-gel trypsin digests followed by MALDI-TOF mass spectrometry at the Leiden University Medical Center (LUMC) as previously described [34].

### Nuclear protein extraction

Nuclear protein extracts from adult male and female schistosomes (~80 parasites/gender) were extracted with the EpiQuik Nuclear Extraction Kit I (Epigentek) according to the manufacturer’s instructions (for tissues). The same technique was used for extraction of nuclear proteins from NIH-3T3 cells. NIH-3T3 cells were cultured to confluency in T75 flasks under the conditions described above, and nuclear protein extraction was performed according to EpiQuik’s instruction for monolayer and adherent cells.

### Assessment of 5mC binding

Induced and “un-induced” rSmMBD2/3-His_6_ soluble protein fractions were dialysed to exchange IMDZ wash buffer for TBS (Tris-buffered saline; 50 mM Tris-HCl, 150 mM NaCl, pH 7.6) prior to assessment of 5mC binding. The 5mC binding activities of induced and “un-induced” rSmMBD2/3-His_6_, nuclear extracts of 7 wk adult male and female parasites and nuclear extracts of NIH-3T3 cells were quantified using the EpiQuik MBD2 Binding Activity Assay Kit (Epigentek) according to the manufacturer’s instruction. Colorimetric readouts of 5mC binding (in the CpG context) were measured by a BMG Labtech Polarstar Omega plate reader. For induced and “un-induced” SmMBD2/3, 5mC binding of 1 μg of soluble protein sample was used (1 μg bovine serum albumin, BSA, was additionally used as a negative control). For nuclear protein extracts, 10 μg of soluble protein was used per sample. Input sample volumes were made up to a total of 3 μl using TBS. As a measure of background, blank samples containing TBS only were used, and this colorimetric reading was subtracted from all other samples. All conditions were set up in triplicate, and results are representative of three replicates derived from single protein samples. For statistical analysis, one-way ANOVA and Tukey’s HSD test were used.

### SmMBD2/3 yeast-2-hybrid identification of SmCBX binding partner

A yeast two-hybrid (Y2H) library was synthesised using the BD Matchmaker Library Construction and Screening kit (Clontech, UK) according to manufacturer instructions. Double-stranded cDNA for the library was made from 2 μg of mixed sex, adult *S*. *mansoni* RNA (isolated from parasites using TRIzol, Invitrogen). The library was constructed by transforming competent *Saccharomyces cerevisiae* (strain AH109) with double-stranded cDNA and the pGADT7-Rec plasmid and transformants were selected on SD/-Leu plates, harvested and stored in 1 ml aliquots at -80°C. The full length SmMBD2/3 coding sequence (GenBank: HM991455.1) was cloned into the Gal4-BD fusion vector, pGBKT7, and expressed within *S*. *cerevisiae* strain Y187. Production of the cDNA library, transformations, toxicity tests, auto-activation tests, mating procedures and the Y2H screen were performed according to the Matchmaker Library Construction & Screening Kits User Manual (Clontech, UK). Toxicity and auto-activation tests of expressed SmMBD2/3 Gal4-AD fusion proteins were performed in both AH109 and Y187 strains and found to be negative. The Y2H screen was performed on SD plates lacking Tryptophan, Leucine, Histidine and Adenosine plus X-α-gal (4 μg/ml) (SD SD/-Trp/-Leu/-His/-Ade + X-α-gal). Positive colonies were subsequently screened for LacZ reporter gene activity using the X-β-gal filter assay (Matchmaker Library Construction & Screening Kits User Manual, Clontech, UK). X-β-gal filter assay colonies were visually categorised according to the intensity of blue, and those with the highest intensity had cDNA library Gal4-AD fusion interacting partners identified by PCR, as described in the Matchmaker Library Construction & Screening Kits User Manual (Clontech, UK). Each in-frame, identified interacting partner was retested by co-transformation into the Y2HGold strain (Clontech, UK) and plating on SD/-Trp/-Leu/-His/-Ade + X-α-gal with additional Aureobasidin A (60 ng/ml). Positive (p53 + LgT) and negative controls (LamC + LgT) were also produced and used as references in the screen.

### Quantification of the SmMBD2/3 and SmCBX interaction strength

The pellet X-β-gal (PXG) assay, as described by Möckli *et al*. [35], was used to quantify SmMBD2/3 + SmCBX interactions. Appropriate negative (pGBKT7 + SmCBX-Gal4-AD, SmMBD2/3-Gal4-BD + pGADT7, pGBKT7 + pGADT7, LgT + LamC) and positive (LgT + p53) controls were produced in the Y2HGold strain (Clontech, UK) and assayed alongside SmMBD2/3 + SmCBX samples. Three colonies of each Y2HGold strain produced were assayed per interacting partner. The plate was scanned using a GS-800 calibrated densitometer and Quantity One (v4.6) software (Biorad, UK). The pixel intensity of each well was quantified using ImageJ. The average pixel intensity value of the LgT + LamC negative controls was used to blank all other samples. Pixel intensities were expressed as a percentage of the average positive control LgT + p53 value. One-way ANOVA and Tukey’s HSD test were used for statistical analysis.

### *Smcbx* transcription profile

Data from the 37,632 element *S*. *mansoni* long-oligonucleotide DNA microarray studies of Fitzpatrick *et al*. [36] was interrogated to find the expression profile of *Smcbx* across 15 different lifecycle stages. Raw and normalised fluorescent intensity values are available via Array Express under the experimental accession number E-MEXP-2094.

### Fluorescence *in situ* hybridisation (FISH) of *Smmbd2/3*, *Smcbx* and *Smh2b* in adult worms

Parasite fixation, permeabilisation, and whole mount fluorescence *in situ* hybridisation (FISH) were performed as previously described [37]. To detect hybridisation signals, Tyramide Signal Amplification was employed using methods previously described [38].

### RNAi interference (RNAi)

Following the perfusion of 7-week infected mice, adult worms were recovered and RNAi performed as previously described [14]. *Smmbd2/3*, *Smcbx* and non-specific *Luciferase (Luc)* siRNA duplexes were purchased from Sigma (siRNA sequences defined in S1 Table). Briefly, 10 adult females or 5 worm pairs were transferred to 4mm electroporation cuvettes containing DMEM (5.4 g/L D-Glucose, Sigma) supplemented with 2 mM L-glutamine, 10,000 Units/ml penicillin and 10,000 μg/ml streptomycin. siRNA duplexes (5 μg) were subsequently added and worms were electroporated with a single pulse at 125V for 20ms using a ECM-830 Square Wave Porator (BTX). For double knockdowns, 5 μg of each *Smmbd2/3* and *Smcbx* siRNA duplex was used and compared to 10μg of si*Luc* duplexes. Mixed sex adult worms (for knockdown assessment by quantitative reverse transcription PCR, qRT-PCR) and adult females (for stem cell quantification) were cultured at 37°C in DMEM (5.4 g/L D-Glucose, Sigma) supplemented with 10% fetal calf serum, 2 mM L-glutamine, 10,000 Units/ml penicillin and 10,000 μg/ml streptomycin in an atmosphere of 5% CO_2_ with a 70% media exchange performed every 24 hr.

### Quantitative reverse transcription PCR (qRT-PCR)

Following RNAi with si*Smcbx*, si*Smmbd2/3* and si*Luc*, mixed-sex adult worms were incubated for a total of 48 hr before processing them for RNA isolation. Briefly, worms were homogenised using a TissueLyser LT (Qiagen, UK) in TRIzol Reagent (Invitrogen, UK) before isolation of total RNA using the Direct-zol RNA Kit (Epigentek, UK). cDNA was then generated, qRT-PCR performed and data analysed as previously described [36]. qRT-PCR primers are defined in S1 Table.

### Quantification of stem cell populations in adult worms

*In vitro* 5’-ethynyl-2’-deoxyuridine (EdU) labelling was performed as previously described [37]. Briefly, RNAi-manipulated adult females were cultured for six days and pulsed with 10 μM EdU for 24 hr at day six. On day seven, female schistosomes were fixed, stained and prepared for laser scanning confocal microscopy (LSCM) imaging. Anterior regions and ovaries were imaged and used to determine the relative number of EdU-labelled nuclei for treatment as well as control groups. For quantification, LSCM images were acquired using a Leica TCS SP5II confocal microscope and a 40X lens (NA 1.25), accruing a total of 15 sections for each Z-stack. For each Z-stack, the fluorescent intensity of the DAPI and EdU channels were used to calculate the total volume (μm^3^) occupied by each fluorophore using the Surface tool in Imaris v8.2 (Bitplane). The percentage of EdU positive nuclei was calculated by dividing the volume of the EdU channel by the volume of the DAPI channel. To investigate significant differences between the siRNA treatments, a one-way ANOVA followed by Tukey HSD test was performed.

## Results

### *Schistosoma mansoni* methyl-CpG binding domain protein 2/3 (SmMBD2/3) is a nuclear-localised protein capable of binding 5mC

**Fig 1:**
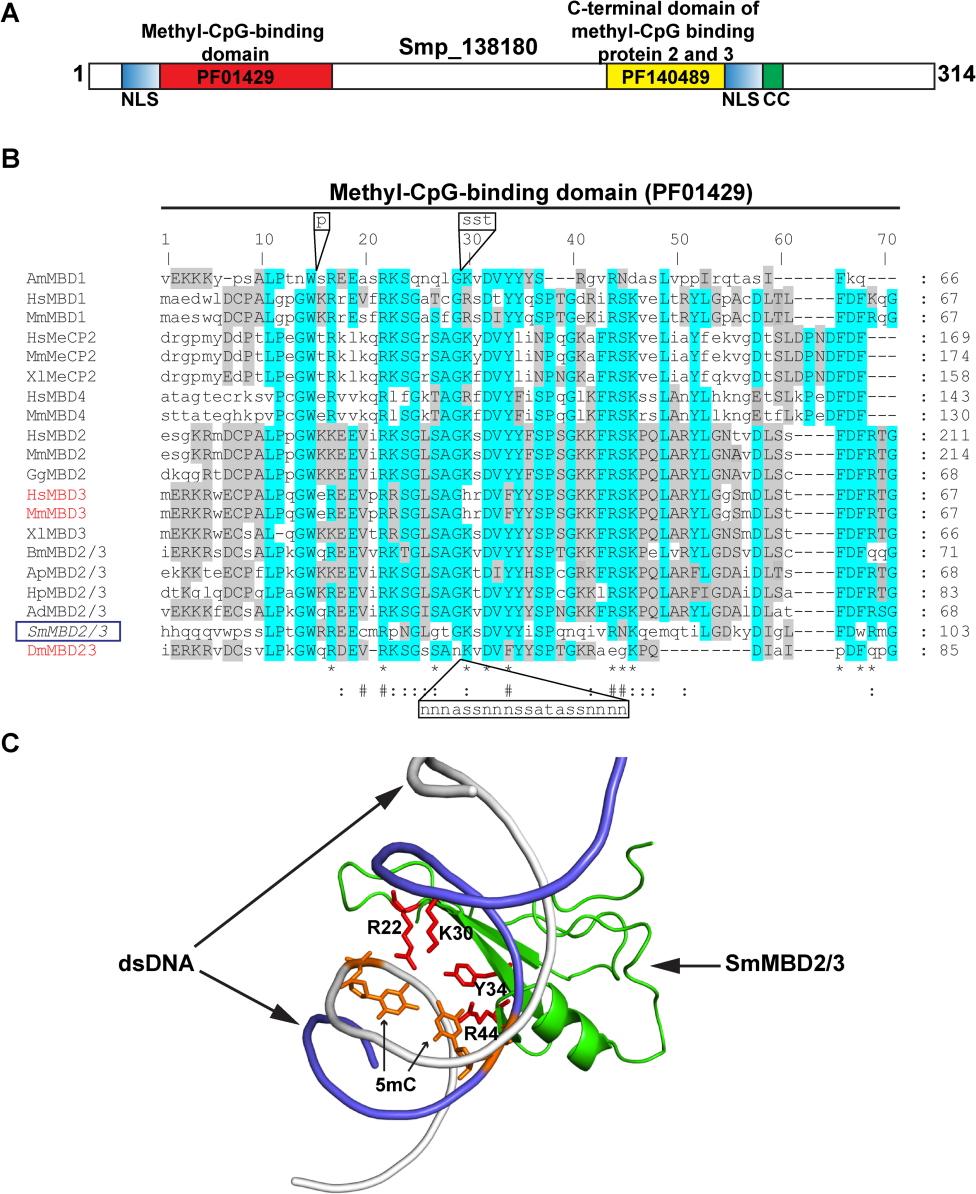
SmMBD2/3 contains amino acid residues critical for binding to 5mC templates. **(A)** Diagrammatic representation of the 314 amino acid SmMBD2/3 (encoded by Smp_138180) illustrating the methyl-CpG (mCpG) binding domain (PF01429), two putative predicted bipartite nuclear localisation signals (NLS), a coiled-coil domain (CC) and a C-terminal domain of methyl CpG binding protein 2 and 3 (PF140489). Amino acid positions are indicated in bold numbering. **(B)** A multiple sequence alignment of the methyl-CpG (mCpG) binding domains (PF01429) collected from SmMBD2/3 (italics and contained in a blue box) and MBD homologs was generated. MBDs unable to bind 5mC are indicated in red. Highly conserved residues are highlighted in turquoise and moderately conserved residues are shaded grey. A ‘*’ indicates amino acid residues that contribute to 5mC binding as assessed by mutational studies (summarised in Table 1). A ‘#’ signifies additional amino acid residues that directly interact with 5mC and a ‘:’ indicates amino acid residues that interact with the DNA phosphate backbone [21]. Amino acid insertions in AmMBD1 and DmMBD2/3 are indicated in black boxes above and below the alignment, respectively. AmMBD1: XP_003250634.1, HsMBD1: NP_002375.1, MmMBD1: NP_038622.2, HsMeCP2: NP_001104262.1, MmMeCP2: NP_001075448.1, XlMeCP2: NP_001081854.1, HsMBD4: NP_001263201.1, MmMBD4: NP_034904.2, HsMBD2: NP_003918.1, MmMBD2 NP_034903.2, GgMBD2: NP_001012403.1, HsMBD3: NP_001268382.1, MmMBD3: NP_038623.1, XlMBD3: AAD55389.1, BmMBD2/3: XP_004929675.1, ApMBD2/3: ACF05483.1, HpMBD2/3: ACF05485.1, AdMBD2/3: [25], SmMBD2/3: CCD59176.1, DmMBD2/3: NP_731370.1. **(C)** Ribbon representation of SmMBD2/3 homology model (green) depicts 5mC-interacting residues (stick representation: amino acids in red, 5mC in orange) found within PF01429. The SmMD2/3 model was generated using the solved crystal structure of *G*. *gallus* MBD2 co-complexed with methylated DNA as detailed in the *Materials and Methods*. The tetra-amino acid archetypal binding pocket is indicated (red).

Our previous studies suggested that SmMBD2/3 and some, but not all, related platyhelminth MBD2/3 homologs contained structural features critical for 5mC binding and diagnostic for this family [6, 14, 39]. This is also true for other representative eukaryote MBDs (Fig 1).

While these previously described features included a methyl-CpG binding domain (PF01429) and a C-terminal domain of methyl-CpG binding protein 2 and 3 (PF140489), our current examination (using cNLS mapper [40] and WormBase-Parasite [41]) of SmMBD2/3 has additionally revealed the presence of two putative atypical bipartite nuclear localisation signals (^13^QTKRSSYANYGKQPQNSMSGQQPHHHQQ^40^, ^271^PMIKTFIVTDDDIRRQEARVKELRKKLEIA^300^) and a coiled-coil domain (^283^ IRRQEARVKELRKKLEIARKK^303^) (Fig 1A). Further sequence analyses of the methyl-CpG-binding domain (PF01429) within SmMBD2/3 and other MBD proteins highlighted the molecular basis for a proposed difference in functional activity. While variation was found in the conservation of SmMBD2/3 residues likely to be important in DNA binding (Fig 1B, ‘:’), the amino acid residues necessary for 5mC interactions (Table 1) were generally well conserved (9 out of 12 residues being identical, ‘*’ in Fig 1B).

**Table 1:** Amino acid residues involved in 5mC binding found within the methyl-CpG-binding domain (PF01429) of MBD proteins.

Critical Residue^1^	References to mutational studies
R17	[26, 42, 43]
R22	[21, 44–47]
S27	[21]
K30	[21, 26, 46–49]
D32	[21, 46, 47]
Y34	[21, 26, 46–49]
R44	[21, 26, 42–44, 46, 47]
S45	[21, 46, 47]
K46	[21]
F66	[42, 43]
F68	[47]
R69	[21, 26, 42, 43]

^1^Amino acid residues are numbered according to their position within the multiple sequence alignment depicted in Fig 1.

Amongst the differences, semi-conservative (S45N) and non-conservative (S27G) substitutions were observed at 2 out of 3 residues in SmMBD2/3. Of particular importance, however, is the finding that SmMBD2/3 retains K30 and Y34. Together with R22 and R44, K30 and Y34 form a tetra-amino acid archetypal binding pocket well-conserved in all MBD binding proteins shown to interact with 5mC (Fig 1C and Table 1) and suggested a functional role for SmMBD2/3 in the nuclei of schistosome cells.

**Fig 2:**
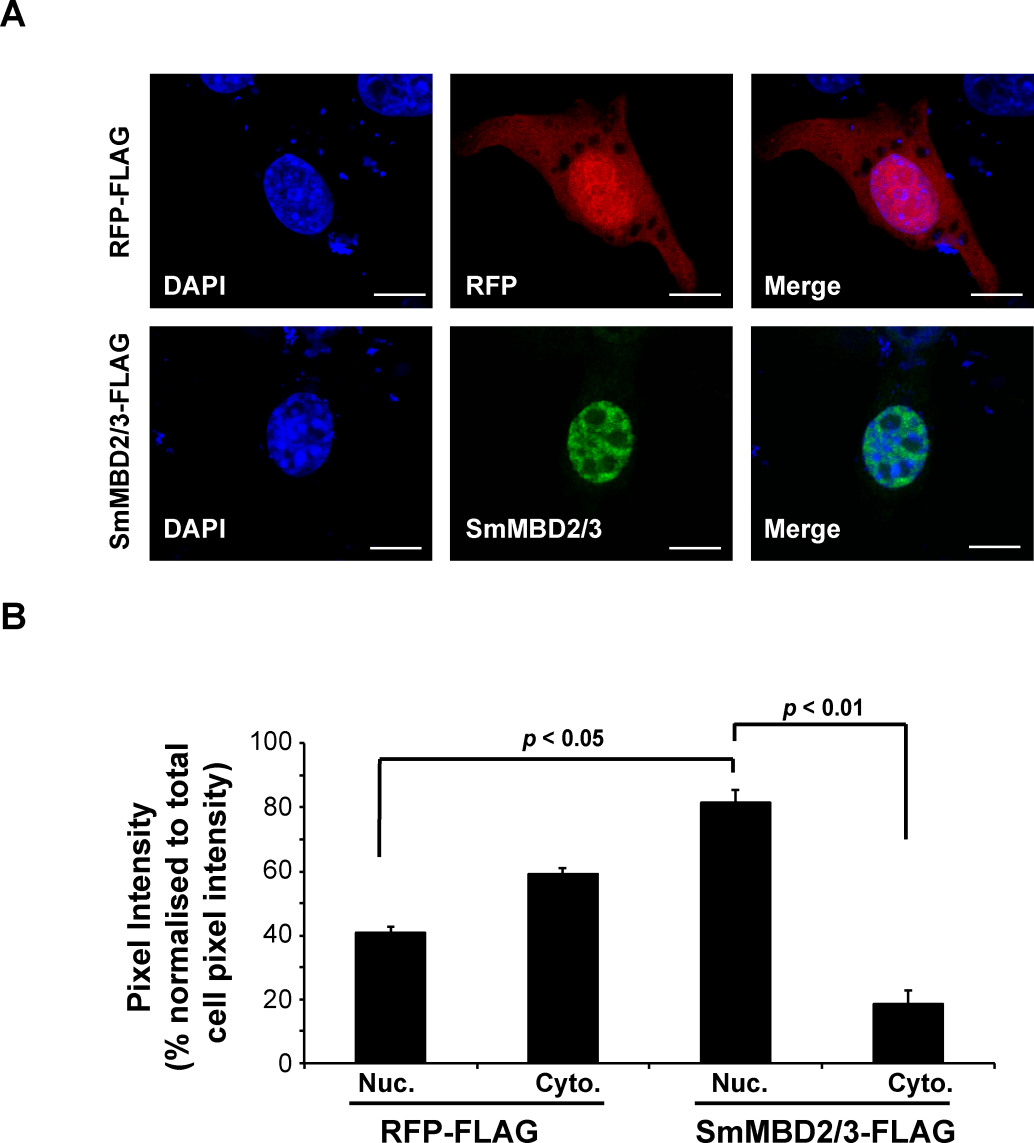
SmMBD2/3 is preferentially localised to nuclei in transfected NIH-3T3 fibroblasts. (A) Representative NIH-3T3 fibroblast cells transfected with full-length SmMBD2/3-FLAG and RFP-FLAG constructs are illustrated. Scale bar = 10 μm. DAPI represents transfected cells visualised with a 405 nm blue diode laser; SmMBD2/3 represents SmMBD2/3-FLAG transfected cells visualised with a 488 nm argon laser; RFP represents RFP-FLAG transfected cells visualised with a 561 nm diode-pumped, solid state laser; Merge represents cellular superimposition of DAPI/SmMBD2/3-FLAG or DAPI/RFP signals. (B) Relative SmMBD2/3-FLAG and RFP-FLAG nuclear (Nuc) vs cytoplasmic (Cyto) fluorescence was quantified from LSCM images collected from a total of 40 transfected cells/construct. SmMBD2/3-FLAG was found to be significantly enriched within nuclear compared to cytoplasmic compartments (*p* < 0.05). SmMBD2/3-FLAG nuclear localisation was also found to be significantly greater than RFP-FLAG nuclear localisation in the transfected cells (*p* < 0.01).

To determine if SmMBD2/3 is capable of translocating to nuclear compartments, transient transfection of a SmMBD2/3-FLAG tagged construct into a surrogate NIH-3T3 *M*. *musculus* fibroblast system was performed and SmMBD2/3 nuclear versus cytoplasmic localisation was quantified (Fig 2).

Here, SmMBD2/3 was found predominantly localised to nuclear compartments of transfected cells in contrast to the more evenly distributed nuclear and cytoplasmic localisation of cells transfected with RFP (representative images, Fig 2A). Quantification of these experiments demonstrated that ~80% of SmMBD2/3 transfected cells contained nuclear-dominated, as opposed to, cytoplasmic-dominated localisation (Fig 2B).

**Fig 3:**
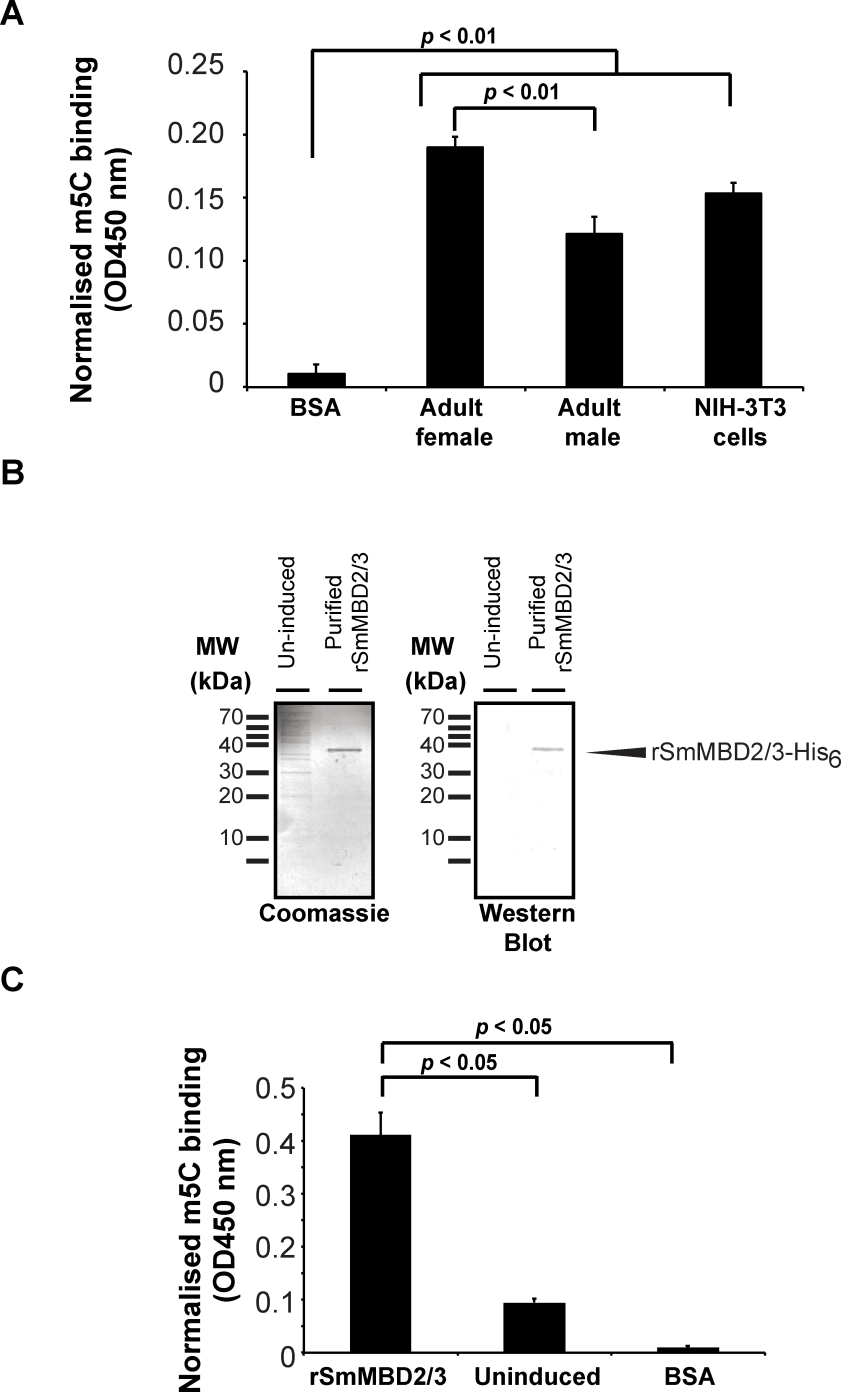
Schistosome nuclear protein extracts and recombinant SmMBD2/3 both contain 5mC binding activities. (A) The 5mC binding capacity of nuclear protein extracts derived from adult male and female worms were quantified using the Epigentek MBD2 binding activity/inhibition assay. NIH-3T3 nuclear protein extracts and BSA were included as positive and negative controls, respectively. A significant difference in 5mC binding (in the CpG context) amongst protein samples was found. (B) IPTG-induced rSmMBD2/3-His_6_ protein (arrowhead; 36.5 kDa) was produced in *E*. *coli*, purified by Ni^2+^-NTA column chromatography and subjected to MALDI-TOF MS (*Materials and Methods*; 22 peptides covering 67% of full length SmMBD2/3 identified). An un-induced sample was also produced and similarly processed. (C) The 5mC binding activity (within a CpG context) of purified rSmMBD2/3-His_6_ was measured using the Epigentek MBD2 binding activity/inhibition assay and compared to un-induced bacterial and BSA protein samples. Significant differences in 5mC binding were observed between rSmMBD2/3-His_6_ and both the BSA and un-induced samples.

Nuclear-dominated localisation of SmMBD2/3 in transient transfected NIH-3T3 cells, as well as our previous description of *Smmbd2/3* biased expression in females (vs males) [14], prompted us to investigate 5mC binding activities in nuclear extracts derived from adult male and female schistosomes (Fig 3).

In both nuclear samples derived from schistosome adults, 5mC binding activity was above background levels (BSA; negative control) and was comparable to that measured in nuclear extracts derived from NIH-3T3 cells (positive control) (Fig 3A). Consistent with *Smmbd2/3*’s female biased expression in adult schistosomes [14], significantly greater 5mC binding activity was detected in female compared to male nuclear extracts (*p* < 0.01). Recombinant expression of SmMBD2/3 (rSmMBD2/3) in *E*. *coli* cells followed by Ni^2+^-NTA purification (Fig 3B) allowed us to directly validate the 5mC binding activity (CpG context) of this nuclear protein. When compared to un-induced rSmMBD2/3 or BSA control samples, purified rSmMBD2/3 demonstrated significantly greater 5mC binding (*p* < 0.05) confirming its role as a functional methyl-CpG-binding protein. rSmMBD2/3 binding to non-methylated DNA targets or to methylated cytosines in diverse nucleotide contexts (i.e. CpA, CpT or CpC) was not assessed.

### SmMBD2/3 interacts with a putative epigenetic adaptor protein SmCBX

**Fig 4:**
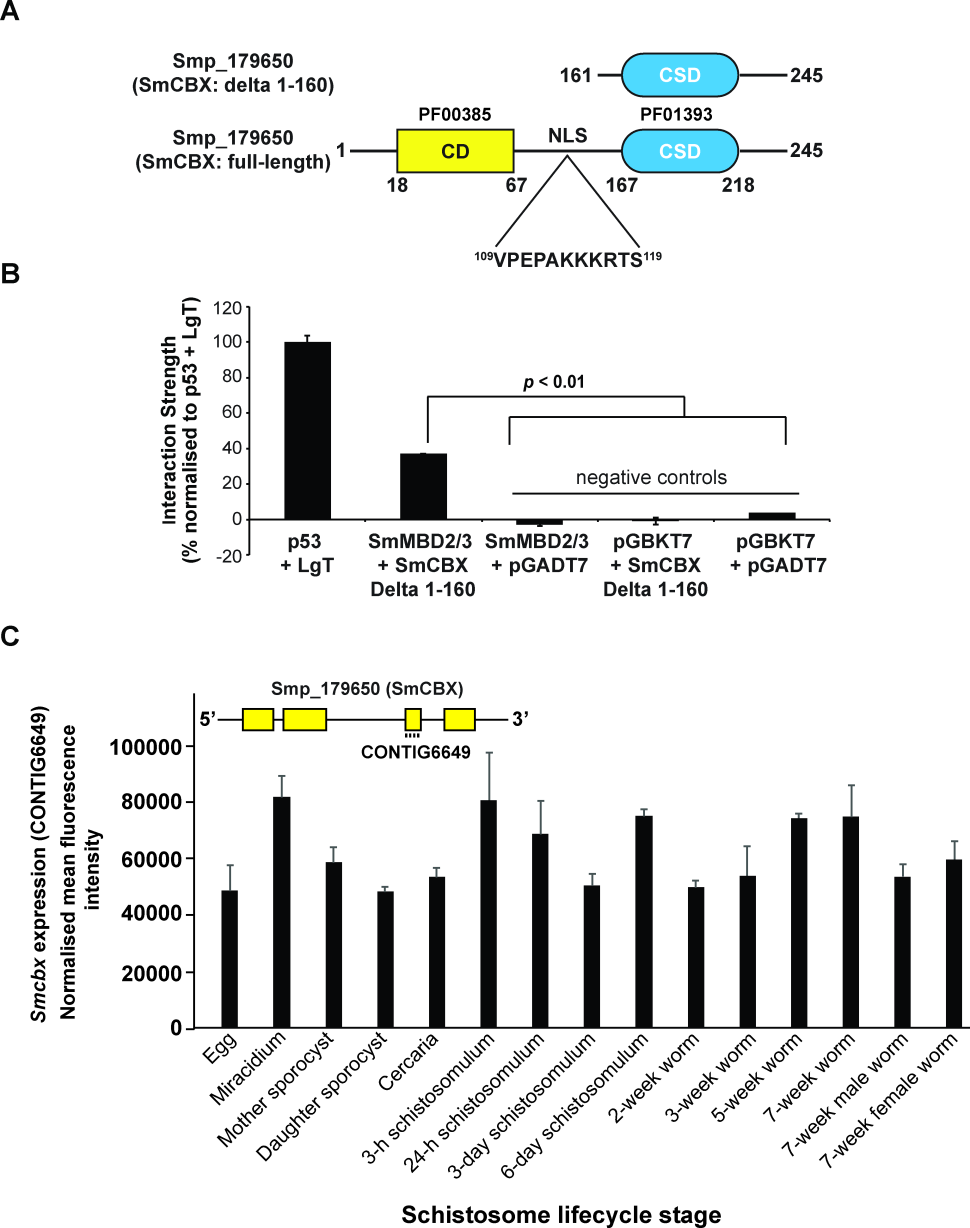
SmMBD2/3 interacts with the nuclear chromobox protein SmCBX (Smp_179650). (A) A truncated version of Smp_179650 (delta 1–160) was repeatedly (5/14 times or 36% of all hits; S2 Table) identified as an interacting partner of SmMBD2/3 in Y2H assays. This truncated version of Smp_179650 contained the chromo shadow domain (CSD; blue oval, PF01393), a region associated with protein-protein interactions [50]. Full-length Smp_179650 also contains the chromodomain (CD; yellow rectangle, PF00385) and a monopartite nuclear localisation signal (NLS, ^109^VPEPAKKKRTS^119^). Amino acid positions are indicated (bold numbers). (B) The SmMBD2/3 –SmCBX (Δ1–160) interaction strength was quantified using the X-β-gal based (PXG) assay [35]. Experimental controls included: p53 + SV40 large T antigen (positive) and SmMBD2/3 + pGADT7 (empty prey vector), pGBKT7 (empty bait vector) + SmCBX/ Δ1–160, pGBKT7 + pGADT7 (all negative). (C) DNA microarray analysis of *Smcbx* expression throughout 15 lifecycle stages. Bar chart represents normalised mean fluorescent intensities + standard deviation (n = 3 replicates/lifecycle stage except adult female, where n = 2) of *Smcbx* transcript abundance derived from oligonucleotide CONTIG6649 as described previously [36]. Inset drawing represents SmCBX (Smp_179650) gene organisation (4 exons–yellow boxes; 3 introns–black lines) and localisation of oligonucleotide CONTIG6649 to exon 3 (SchistoGeneDB v5.2).

SmMBD2/3’s nuclear localisation in a heterologous transfection system and rSmMBD2/3’s binding to 5mC provided further evidence for a functional DNA methylation machinery operating within schistosome parasites [6, 14, 39]. As MBD proteins are recognised ‘readers’ of DNA methyltransferase enzymatic ‘writers’, they importantly serve as an epigenetic bridge between DNA and proteins involved in the formation and regulation of diverse chromatin states [16]. Thus, using Y2H screening of adult schistosome cDNA libraries, we subsequently investigated whether SmMBD2/3 interacted with other known epigenetic regulators or adaptors (Fig 4).

The single most abundant SmMBD2/3-interacting protein identified in these Y2H assays was a C-terminal, in-frame, truncation of Smp_179650 (Fig 4A); this truncation (SmCBX: Δ1–160) was identified five independent times (or 36% of all hits; Y2H results summarised in S2 Table). Full-length Smp_179650 encodes a protein with sequence similarity to epigenetic adaptor heterochromatin- (HP) or chromobox- (CBX) proteins [51, 52] and contains an N-terminal chromodomain (CD; PFAM PF00385, AAs 18–67), a monopartite nuclear localisation signal (NLS; AAs 109–119) and a C-terminal chromo shadow domain (CSD; PFAM PF01393, AAs 167–218). Chromobox CDs are responsible for binding to tri-methylated lysine (K) 9 of histone 3 (H3K9me3) [53, 54] and full-length SmCBX contains all critical AAs within this domain necessary for ‘reading’ H3K9me3 modifications (S1 Fig). Chromobox CSDs are necessary for initiating and maintaining protein-protein interactions (PPIs) [55]; the presence of the CSD within SmCBX (Δ1–160) likely explains all five, Y2H-detected, SmMBD2/3 interactions.

To quantify the interactive strength of CSD-containing SmCBX (Δ1–160) to SmMBD2/3, a modified X-β-gal based assay [35] was performed (Fig 4B). Here, binding of SmMBD2/3 and SmCBX (Δ1–160) was significantly higher than the empty vector negative control (pGBKT7+pGADT7; *p*<0.01). Similar to the negative controls, undetectable reporter expression was observed in yeast cells transfected with either SmMBD2/3 or SmCBX (Δ1–160) constructs alone and confirmed that auto-activation was not responsible for the specific SmMBD2/3-SmCBX (Δ1–160) interaction detected in the Y2H assays. Lifecycle expression profiling demonstrated that *Smcbx* was abundantly transcribed in all schistosome developmental forms analysed (Fig 4C). Together, these data suggest that SmMBD2/3 interacts with the highly abundant epigenetic adaptor protein SmCBX and this interaction is stably maintained by SmCBX’s CSD.

### *Smmbd2/3* and *Smcbx* co-localise to mesenchymal-, proliferating somatic- and germline stem- cells

**Fig 5:**
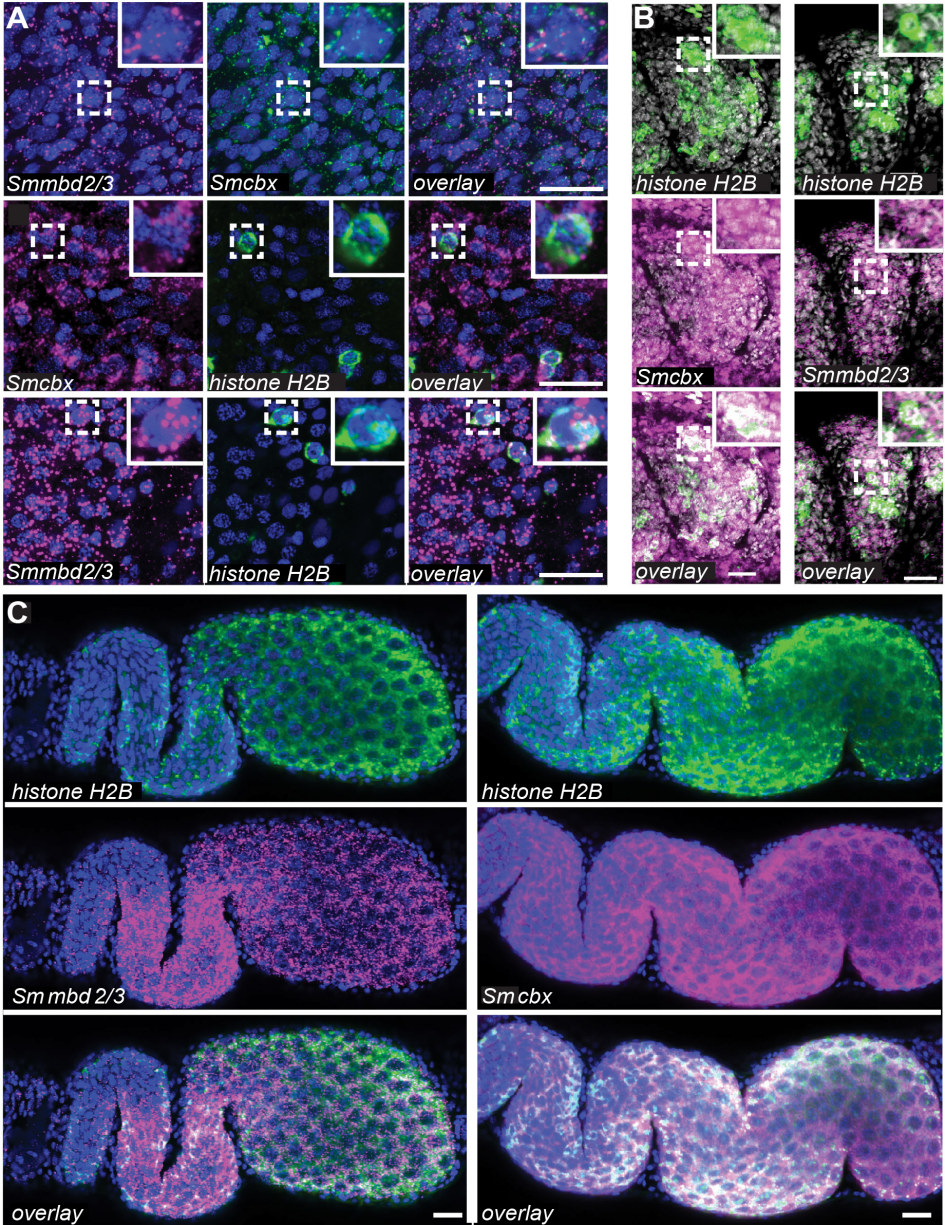
*Smmbd2/3* and *Smcbx* are broadly expressed in male and female schistosomes. (A) Expression of *Smmbd2/3* and *Smcbx* in male somatic tissues, (B) testes and (C) ovaries relative to *Smhistone H2B*. Both genes are broadly expressed in somatic tissues, including the *histone H2B*^+^ neoblasts and in most cell types (*histone H2B*^*-*^) within the male and female germ line. Scale bars = 20 μm. Blue = DAPI. Magenta and green = pseudocoloured antisense RNA probes for *Smmbd2/3*, *Smcbx* and *Smhistone H2B*. White = co-localisation of two RNA probes. Where illustrated, delineated areas (dashed white boxes) are magnified in the insets (solid white boxes).

To provide supportive evidence for the Y2H-identified, SmMBD2/3-SmCBX interactions, localisation of both *Smmbd2/3* and *Smcbx* transcripts in adult schistosomes was explored by fluorescence *in situ* hybridisation (FISH) (Fig 5).

Here, both *Smmbd2/3* and *Smcbx* transcripts were found widely distributed throughout schistosome mesenchymal tissues (Fig 5A). In many (if not all) mesenchymal cells, *Smmbd2/3* and *Smcbx* were spatially co-expressed (Fig 5A, upper row; white boxes). Interestingly, both *Smmbd2/3* and *Smcbx* were also found in a sub-population of mesenchymal cells co-expressing *Smhistone H2B* (Fig 5A, lower two rows; white boxes), a known marker for proliferating neoblasts in adult parasites [37]. Amongst the reproductive tissues, *Smmbd2/3*—*Smhistone H2B* and *Smcbx*—*Smhistone H2B* co-localisation was also broadly expressed in many cells of the male (Fig 5B) and female (Fig 5C) gonads. Supporting the Y2H PPI results (Fig 4), these FISH data provided complementary evidence for SmMBD2/3 and SmCBX interactions in adult schistosomes and demonstrated that both genes were expressed within proliferating (H2B^+^) and differentiated (H2B^-^) cells.

### *Smmbd2/3* and *Smcbx* are required for neoblast, but not ovarian stem cell, proliferation in adult female schistosomes

**Fig 6:**
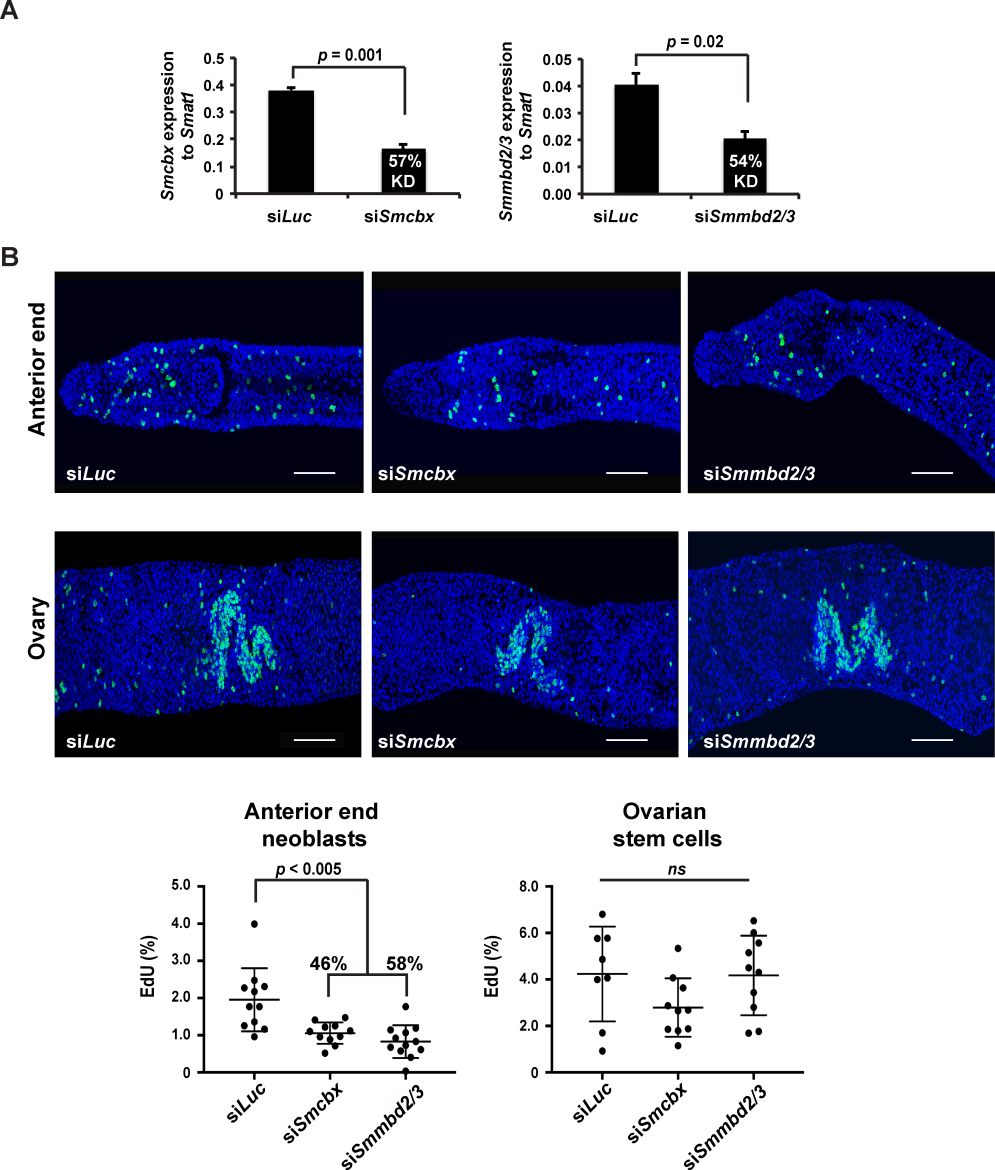
RNAi-mediated knockdown of *Smmbd2/3* and *Smcbx* affects the proliferation of schistosome stem cells. (A) Seven-week old adult male and female schistosomes were electroporated with 5 μg siRNA duplexes targeting luciferase (si*Luc*), *Smcbx* (si*Smcbx*) or *Smmbd2/3* (si*Smmbd2/3*). Following 48 hr, total RNA was harvested and subjected to qRT-PCR. Percent knockdown (KD) and statistical significance (Student’s *t* test, two tailed, unequal variance) is indicated. All siRNA and qRT-PCR DNA sequences are included in S1 Table. (B) Representative anterior ends and ovaries of female schistosomes treated with siRNA duplexes at day seven post treatment. Blue = DAPI; Green = EdU^+^ cells. Bar = 50 μM. Column scatter plot (horizontal bars = mean and +/- StDev of mean) represents the percentage of proliferating cells remaining in female worms treated with siRNA duplexes for seven days (si*Luc*, n = 11; si*Smcbx*, n = 11; si*Smmbd2/3* = 12). The percentage of proliferating cells affected by knockdown (in comparison to *siLuc* control worms) is indicated where significant (one-way ANOVA followed by Tukey HSD test).

RNAi was subsequently used to investigate the function of *Smmbd2/3* and *Smcbx* in adult worms (Fig 6).

Here, siRNAs targeting either *Smmbd2/3* or *Smcbx* in adult worm pairs led to a greater than 50% reduction in transcript abundance when compared to control worms (57% for si*Smcbx* treated worm pairs, 54% for si*Smmbd2/3* treated worm pairs) (Fig 6A). Together, these data confirmed that RNAi could reduce the pools of *Smmbd2/3* and *Smcbx* in adult schistosomes.

As our FISH results showed co-localisation of *Smmbd2/3* and *Smcbx* to schistosome neoblasts and reproductive tissues (Fig 5), we next investigated whether either *Smmbd2/3* or *Smcbx* knockdown could affect aspects of schistosome stem cell biology. Adult females were chosen for these experiments due to greater *Smmbd2/3* expression [14] and 5mC binding (Fig 3) found in this gender compared to males. In either *Smmbd2/3* or *Smcbx* knockdown conditions, adult females contained noticeably fewer EdU^+^ somatic cells (58% and 46% less, respectively, to control si*Luc* treated worms) throughout their bodies compared to controls (representative anterior regions; Fig 6B). In contrast, ovarian stem cell proliferation was not significantly affected by *Smmbd2/3* or *Smcbx* knockdown. Females treated with siRNAs targeting both *Smmbd2/3* and *Smcbx* (double knock-down) showed a similar neoblast deficiency phenotype (60% less EdU^+^ somatic cells compared to control si*Luc* treated worms, S2 Fig).

### Schistosome oviposition is dependent upon both *Smmbd2/3* and *Smcbx*

**Fig 7:**
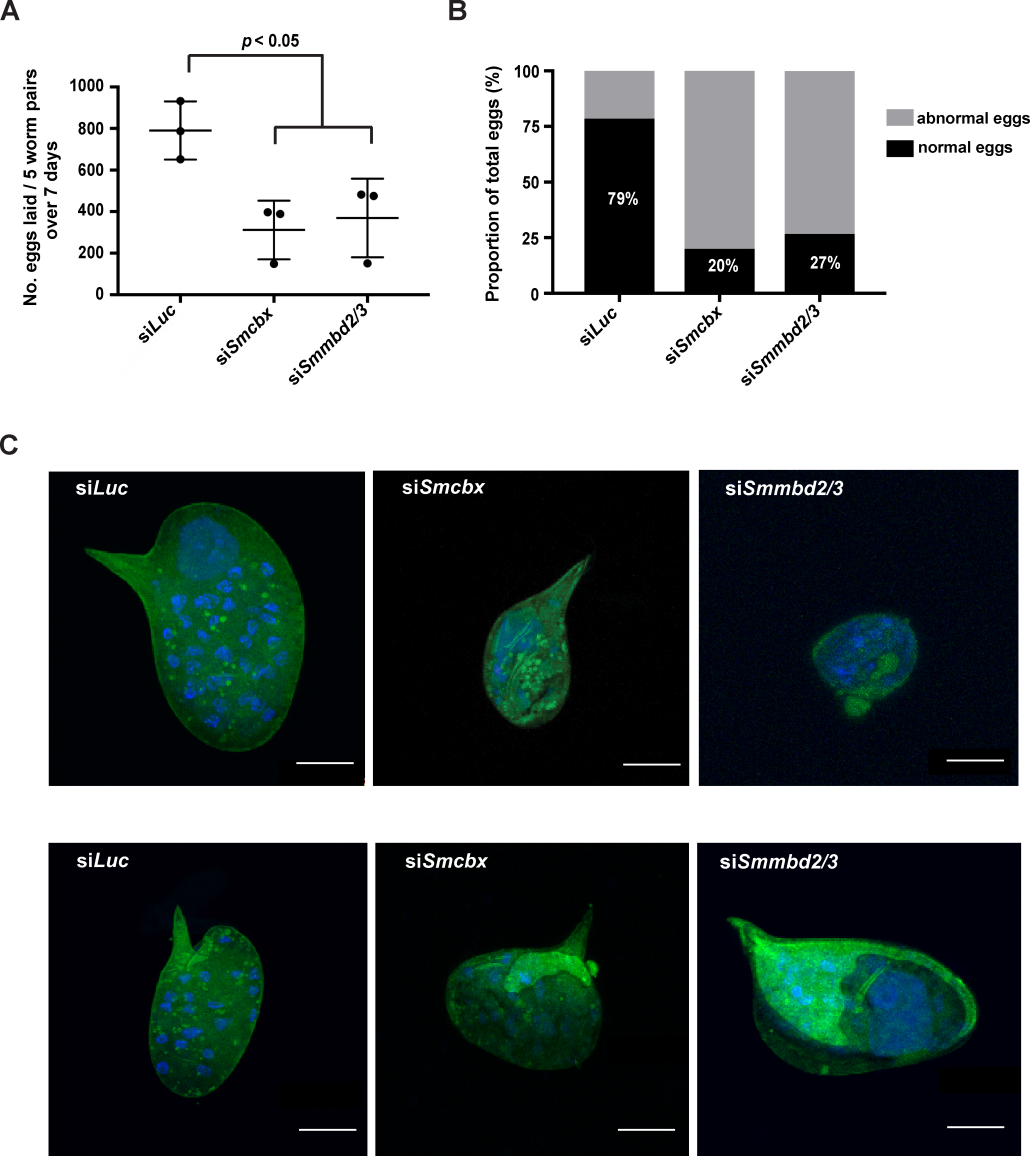
SmMBD2/3 and SmCBX are both required for schistosome oviposition. (A) Seven-week old adult male and female schistosome pairs were electroporated with 5 μg siRNA duplexes targeting luciferase (si*Luc*), *Smcbx* (si*Smcbx*) or *Smmbd2/3* (si*Smmbd2/3*). At day 7 post treatment, eggs were collected from wells (5 worm pairs/well; n = 3) and counted. A one-way ANOVA followed by Tukey HSD test was performed to identify statistically significant treatments. (B) Percentage of eggs (n = 14 for si*Luc*, n = 20 for si*Smcbx*, n = 15 for si*Smmbd2/3*) demonstrating abnormal (grey) versus normal (black) phenotypes. Normal = oval eggs with lateral spine, containing regular surface autofluorescence. (C) Representative fluorescent images of eggs collected from wells of si*Luc*, si*Smcbx* and si*Smmbd2/3* treated worm pairs. Green = eggshell autofluorescence; blue = DAPI^+^ cells. Bar = 20 μm.

As a defect in neoblast proliferation was observed in adult females treated with either *Smmbd2/3* or *Smcbx* siRNAs, other gross phenotypic abnormalities were additionally sought in these *in vitro* manipulated schistosomes (Fig 7).

Here, despite an incomplete reduction in *Smcbx* or *Smmbd2/3* transcript levels (Fig 6A), a significant decrease in the number of normal (oval, containing a lateral spine with regular surface autofluorescence) schistosome eggs was consistently observed in siRNA treated parasites compared to si*Luc* controls (Fig 7A). This decrease in oviposition of normal eggs was also associated with the increased production of abnormal eggs (Fig 7B). Noticeable phenotypes observed in both treatment (si*Smcbx* and si*Smmbd2/3*) conditions included exemplars without lateral spines, individuals demonstrating reduced egg volumes and entities containing irregular autofluorescence (Fig 7C). Regardless of siRNA treatment, and despite these gross morphological differences, vitellocytes (DAPI^+^ cells) were present in all eggs examined (Fig 7C).

## Discussion

Schistosome development is influenced by interactions with three distinct niches (freshwater ecosystem, snail intermediate host and mammal definitive host) and is molecularly controlled by genetic as well as epigenetic processes [6, 36]. While schistosomes do not harbour the extreme developmental plasticity potential exhibited by nematodes [56], their ability to remain responsive to diverse environmental signals assists in the establishment of heritable variations critical for infection success [57]. Therefore, elucidating how schistosome epigenetic components cooperatively regulate key parasitological processes and shape heritable traits will likely uncover new targets for schistosomiasis control. Here, we provide evidence for the role of SmMBD2/3 and SmCBX in the biology of schistosome somatic stem cells (neoblasts) and additionally suggest that pharmacological disruption of these interacting partners will lead to defects in the most important aspect of schistosome mediated pathology and lifecycle maintenance, egg production.

While not every eukaryotic MBD binds to methylated DNA (reviewed in [58]), our data indicate that SmMBD2/3 contains the necessary features responsible for 5mC recognition (Fig 1), nuclear localisation (Fig 2) and functional 5mC binding (Fig 3). These particular findings are in contrast to those obtained from a detailed study of the only other platyhelminth MBD protein characterised to date, SmedMBD2/3 [20]. In this previous study, Jaber-Hijazi *et al*. demonstrated that SmedMBD2/3’s function in planarian tissue homeostasis was independent of 5mC binding [20]. The most likely explanation for these differential results between related platyhelminth MBD homologs is amino acid substitutions of critical 5mC-binding residues in the SmedMBD2/3 MBD domain (also observed in both HsMBD3 and MmMBD3, Fig 1 and Table 1), which are well-conserved in SmMBD2/3 [39]. MBD sequence divergence, along with variable levels of detectable DNA methylation (detectable levels found in *S*. *mansoni* [14, 15], undetectable levels found in *S*. *mediterranea* [20]), strongly suggests that the core platyhelminth DNA methylation machinery (MBDs and DNMTs) is diversely utilised within this group of animals and may be involved in other nuclear functions in addition to or in replacement of ‘reading’ DNA methylation marks [14] (Fig 3). For example, human MBD1 can bind to unmethylated cytosines via a CxxC domain as well as 5mC via its MBD [59]. Similarly, human MBD4 has an additional 5mC binding function, DNA repair, and this particular activity is facilitated by the presence of a DNA glycosylase domain [60]. Finally, while HsMBD5 and HsMBD6 both contain a MBD, they do not bind to 5mC; the presence of PWWP domains (present in MBD5) and P-rich domains (located in both MBD5 and MBD6) likely defines their role in other biological activities [61]. While none of these motifs are present in SmMBD2/3, a C-terminal coiled-coil region is clearly identifiable (Fig 1C). As Tatematsu *et al*., demonstrated that coiled-coil regions are essential for homo-dimerisation of HsMBD2 [62], the presence of this C-terminal domain within SmMBD2/3 is likely responsible for PPIs important for regulating higher-order chromatin structure in schistosome nuclei. Evidence to support this contention was derived from our Y2H studies confirming that SmMBD2/3 specifically interacts with an epigenetic adaptor protein SmCBX (Fig 4).

Chromobox (CBX) proteins (also known as heterochromatin protein 1; HP1) are non-histone, chromatin-interacting proteins involved in the regulation of heterochromatin, transcription and development [52]. Previous studies have demonstrated that both HsMBD1 and HsMeCP2 interact with HsHP1; the consequence of these interactions results in heterochromatin formation and transcriptional repression [51, 63]. Our results, therefore, are in line with these previous reports and illustrate that schistosomes maintain this conserved molecular interaction (Fig 4). As SmCBX’s CD contains all of the features necessary for H3K9me3 (a transcriptionally repressive histone mark; [64]) interactions (S1 Fig) and SmCBX’s CSD is associated with SmMBD2/3 binding (Fig 4A and 4B), this protein complex (along with other, yet to be identified proteins) is well-positioned to link the epigenetic processes of schistosome DNA methylation and post-translational histone modifications. Therefore, this data provides the first evidence within the Platyhelminthes that epigenetic cross-talk can occur and may have particular relevance in the context of schistosome chromatin structure, genome function and phenotypic manifestations. Further investigations exploring how SmMBD2/3-SmCBX interactions shape or modulate parasite developmental processes or transcriptional regulation could reveal novel (epigenetically-directed) strategies for anti-schistosomal control.

As a first step towards this goal, we investigated the spatial distribution of both *Smmbd2/3* and *Smcbx* within adult schistosomes and found co-localisation to mesenchymal cells (*histone H2B-*) as well as *histone H2B*^*+*^ germ line cells and somatic neoblasts (Fig 5). While the function of the SmMBD2/3-SmCBX protein complex in schistosome mesenchymal cells was not explored, their importance in stem cell biology was investigated due to this cell population’s role in adult schistosome development and host interactions [37, 65]. Here, RNAi-mediated knockdown of either *Smmbd2/3* or *Smcbx* (or both *Smmbd2/3* and *Smcbx*, S2 Fig) in adult schistosomes led to significant reductions in the numbers of proliferative neoblasts, but not ovarian stem cells (Fig 6). This discrepancy (affecting neoblast but not ovarian stem cell proliferation) may be due to: 1) additional function (s) associated with non-5mC-mediated DNA binding [58, 59], 2) capacity to form other multi-protein complexes [58], 3) differential role (s) in neoblast/ovarian stem cells or 4) the incomplete knockdown of both *Smmbd2/3* (54%) and *Smcbx* (57%) transcript levels in female schistosomes (Fig 6A). As ovarian stem cells appear to contain greater quantities of both *Smmbd2/3* and *Smcbx* compared to neoblasts (Fig 5C vs 5A and [66]), residual levels of these two epigenetic regulators after RNAi may be sufficient to maintain proliferation in ovarian stem cells, but not neoblasts. Nevertheless, partial depletion of *Smmbd2/3* or *Smcbx* transcript pools both significantly affected schistosome egg production and phenotype (Fig 7). While vitellocyte production in si*Smmbd2/3* or si*Smcbx* treated females was comparable to si*Luc* controls (all normal/abnormal eggs contained DAPI^+^ vitellocytes, Fig 7C), this cell population’s role in egg-shell tanning appeared altered (noticeable difference in autofluorescence were observed). In addition to tanning, the size and shape of *in vitro* laid eggs would suggest that deficiencies in *Smmbd2/3* and *Smcbx* also affect ootype and Mehlis’ gland contributions to oviposition. Therefore, further studies are necessary to understand how *Smmbd2/3* and *Smcbx* contribute to vitellaria as well as Mehlis’ gland function, egg production rates, ootype biology and normal egg-shell tanning. However, similar egg-laying defects were observed in parasites treated with the DNA methylation inhibitor 5-AzaC [14], which, together with our current findings, provides further evidence for a functionally relevant DNA methylation machinery in schistosomes.

Our RNAi results are also consistent with studies conducted in mammalian systems where knockdown of *Cbx2*, *Cbx3*, *Cbx4* or *Cbx8* all resulted in decreased stem cell proliferation [67–70]. Where studies have been conducted in non-parasitic platyhelminth species (i.e. planarians), critical roles have also been established for both MBD2/3 and CBX in neoblast function [20, 71, 72]. However, due to differences (sometimes undetectable [20]) in underlying levels of genome methylation amongst platyhelminth species [39], the function of MBD2/3 and CBX proteins within the phylum is also likely to differ. For example, while SmMBD2/3 displays ubiquitous spatial expression throughout adult schistosomes (Fig 5), SmedMBD2/3 is exclusively expressed in planarian proliferating ASCs and germ line cells only [20]. Additionally, and also in contrast to our results where SmMBD2/3 appears vital for schistosome neoblast (but not ovarian stem cell) proliferation (Fig 6B), planarian neoblast proliferation does not seem to involve SmedMBD2/3 [20]. These data, together with those indicating a role for SmedCBX1 in regulating planarian neoblast function [71], strongly support differing functions of MBD2/3-CBX protein complexes within the platyhelminths. Indeed, characterising the differing functions of related platyhelminth epigenetic components (amongst the backdrop of divergent DNA methylomes) represents an exciting area of future research into the evolution of this phylum and control of its parasitic species [6].

Together, our data provides growing evidence supporting the view that schistosomes have an intact DNA methylation machinery (SmDNMT2 [14] and SmMBD2/3, this study). A suggestive link between DNA methylation and post-translational histone modifications (mediated by SmMBD2/3-SmCBX interactions) also indicates that the wider schistosome epigenetic pathway operates similarly to other characterised eukaryotes containing measurable DNA methylomes. Where differences in the roles ascribed to platyhelminth epigenetic components do occur, it is likely that these are related to loss of gene function (mutations in core components), lack of DNA methylation, divergent developmental biology pathways (free-living or parasitic species) or a combination of all three. The molecular details as to how SmMBD2/3-SmCBX interactions modify chromatin, influence other PPIs, regulate neoblast proliferation or shape other aspects of schistosome genome/transcriptome biology leading to egg production defects awaits further investigations. The results of such studies will lead to a greater understanding into how schistosome epigenetic components shape the developmental biology of this pathogen responsible for a devastating neglected infectious disease and perhaps shed light on novel ways for controlling its public health significance.

## Supporting information

S1 Table Reverse transcription quantitative real time PCR (qRT-PCR) and small interfering RNA (siRNA) oligonucleotide sequences used in this study. (XLSX)

S2 Table SmMBD2/3 interacting proteins identified in this study. (XLSX)

S1 Fig Alignment of the SmCBX chromo domain (CD) to other CD-containing proteins reveal conservation of critical residues required for H3K9me3 binding. The chromo domain (CD) from *Homo sapiens* Suv39h1, labelled HsSUV39H1 in the alignment (GenBank accession number: CAG46546) and *Drosophila melanogaster* HP1, labelled DmHP1 in the alignment (GenBank accession number: ACI96784.1) were aligned against that of SmCBX. Invariant residues are highlighted in blue and presented in upper case. Partially conserved residues (i.e. among pairs with similar physico-chemical properties) are shaded grey and presented in upper case. Non-conserved residues are shown in lower case and are not shaded. Residues known to form the aromatic cage that interacts with the methylammonium group of H3K9me3 are indicated below by “*”. Residues indicated by “#” are involved in direct interactions and recognition of histone H3 [53, 54].
(DOCX)

S2 Fig RNAi-mediated knockdown of both *Smmbd2/3* and *Smcbx* affects the proliferation of schistosome stem cells. (A) For double knockdown experiments, seven-week old adult male and female schistosomes were electroporated with 5 μg siRNA duplexes targeting *Smcbx* (si*Smcbx*) and *Smmbd2/3* (si*Smmbd2/3*); 10μg of si*Luc* duplexes was used as the negative control. Following 48 hr, total RNA was harvested and subjected to qRT-PCR. Percent knockdown (KD) and statistical significance (Student’s *t* test, two tailed, unequal variance) is indicated. All siRNA and qRT-PCR DNA sequences are included in S1 Table. (B) Representative anterior ends of female schistosomes treated with siRNA duplexes at day seven post treatment. Blue = DAPI; Green = EdU^+^ cells. Bar = 50 μM. (C) Bar chart (+/- StDev of mean) represents the percentage of proliferating cells remaining in female worms treated with siRNA duplexes for seven days (si*Luc*, n = 4; si*Smcbx* & si*Smmbd2/3* = 6). Statistical significance is indicated (Student’s *t* test, two tailed, unequal variance).
(PDF)
