# Supplementary material for: Methyl-CpG-binding (SmMBD2/3) and chromobox (SmCBX) proteins are required for neoblast proliferation and oviposition in the parasitic blood fluke Schistosoma mansoni
Source: PLoS Pathog. 2018 Jun 28;14(6):e1007107. doi: 10.1371/journal.ppat.1007107 (PMC6023120; doi:10.1371/journal.ppat.1007107)
Supplement: S1 Fig — The chromo domain (CD) from Homo sapiens Suv39h1, labelled HsSUV39H1 in the alignment (GenBank accession number: CAG46546) and Drosophila melanogaster HP1, labelled DmHP1 in the alignment (GenBank accession number: ACI96784.1) were aligned against that of SmCBX. Invariant residues are highlighted in blue and presented in upper case. Partially conserved residues (i.e. among pairs with similar physico-chemical properties) are shaded grey and presented in upper case. Non-conserved residues are shown in lower case and are not shaded. Residues known to form the aromatic cage that interacts with the methylammonium group of H3K9me3 are indicated below by “*”. Residues indicated by “#” are involved in direct interactions and recognition of histone H3 [53, 54]. (DOCX) [file ppat.1007107.s003.docx]

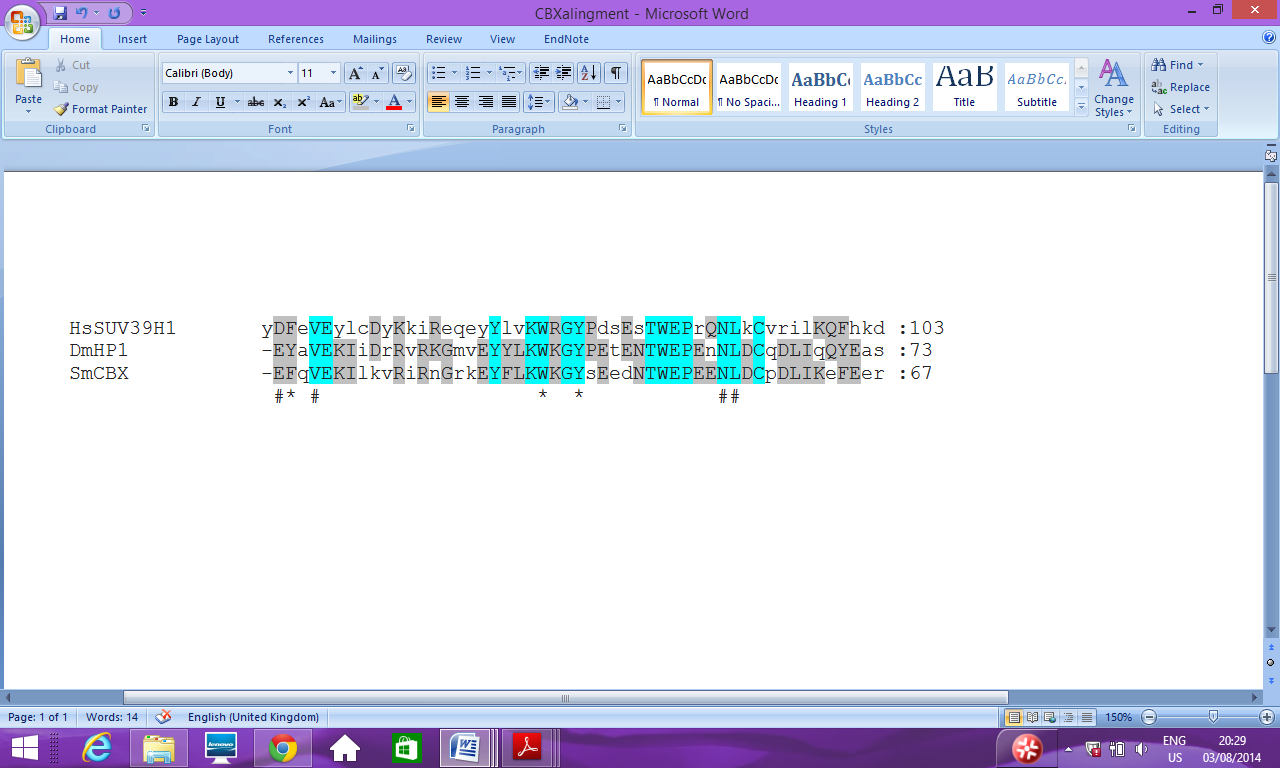


**S1 Figure. Alignment of the SmCBX chromo domain (CD) to other CD-containing proteins reveal conservation of critical residues required for H3K9me3 binding.** The chromo domain (CD) from *Homo sapiens* Suv39h1, labelled HsSUV39H1 in the alignment (GenBank accession number: CAG46546) and *Drosophila melanogaster* HP1, labelled DmHP1 in the alignment (GenBank accession number: ACI96784.1) were aligned against that of SmCBX. Invariant residues are highlighted in blue and presented in upper case. Partially conserved residues (i.e. among pairs with similar physico-chemical properties) are shaded grey and presented in upper case. Non-conserved residues are shown in lower case and are not shaded. Residues known to form the aromatic cage that interacts with the methylammonium group of H3K9me3 are indicated below by “*”. Residues indicated by “#” are involved in direct interactions and recognition of histone H3 [55, 56].
