# Supplementary figures and images for: Methyl-CpG-binding (SmMBD2/3) and chromobox (SmCBX) proteins are required for neoblast proliferation and oviposition in the parasitic blood fluke Schistosoma mansoni
Source: PLoS Pathog. 2018 Jun 28;14(6):e1007107. doi: 10.1371/journal.ppat.1007107 (PMC6023120; doi:10.1371/journal.ppat.1007107)

A

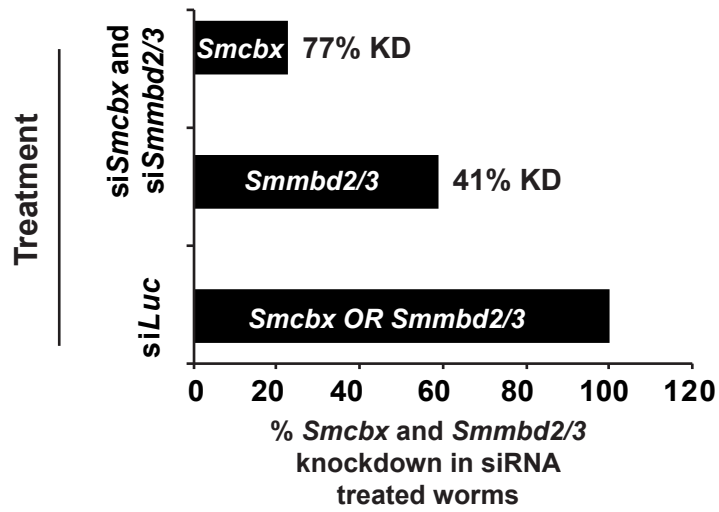

B

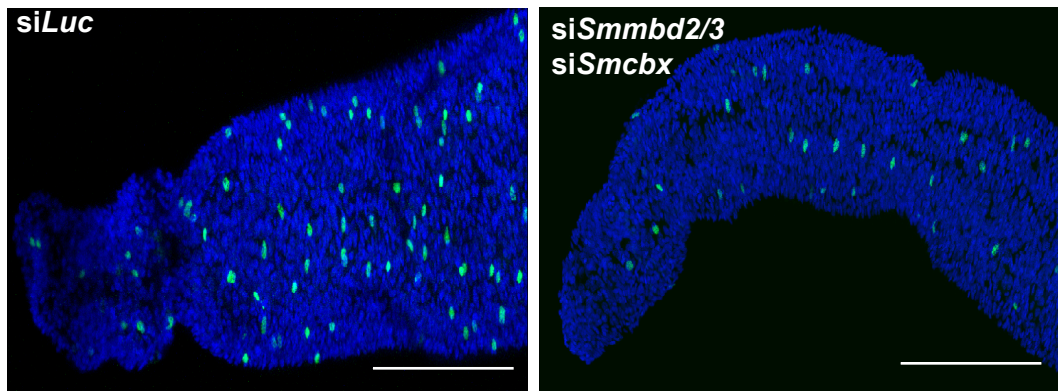

C

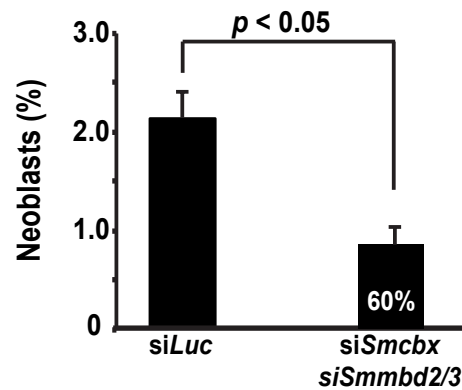

Supplement: S2 Fig — (A) For double knockdown experiments, seven-week old adult male and female schistosomes were electroporated with 5 μg siRNA duplexes targeting Smcbx (siSmcbx) and Smmbd2/3 (siSmmbd2/3); 10μg of siLuc duplexes was used as the negative control. Following 48 hr, total RNA was harvested and subjected to qRT-PCR. Percent knockdown (KD) and statistical significance (Student’s t test, two tailed, unequal variance) is indicated. All siRNA and qRT-PCR DNA sequences are included in S1 Table. (B) Representative anterior ends of female schistosomes treated with siRNA duplexes at day seven post treatment. Blue = DAPI; Green = EdU+ cells. Bar = 50 μM. (C) Bar chart (+/- StDev of mean) represents the percentage of proliferating cells remaining in female worms treated with siRNA duplexes for seven days (siLuc, n = 4; siSmcbx & siSmmbd2/3 = 6). Statistical significance is indicated (Student’s t test, two tailed, unequal variance). (PDF) [file ppat.1007107.s004.pdf]
